# Arkansas State Medical Association

**Published:** 1877-04-20

**Authors:** 


### ARKANSAS STATE MEDICAL ASSOCIATION.

It would appear from a circular sent us by the Secretary of the *Arkansas State Medical Association*, that the American Medical Association, at its last meeting, excommunicated that body, recognizing in its stead a later organization known as the *State of Arkansas Medical Society*. From the vigor and earnestness with which the facts and grievances are set forth in this circular, it is evident that the controversy between the two Societies has become very animated and exciting.

If the statements contained in this circular can be satisfactorily established at the next meeting of the American Medical Association, thereby showing the grounds of their previous ruling to have been unfounded, and their action hasty and unjust, it would seem incumbent upon that body to retrace its steps, and take such action as will be impartial, and in strict conformity with truth and justice.

